# Pulmonary hypertension: the importance of a multidisciplinary approach

**DOI:** 10.1007/s12471-016-0848-0

**Published:** 2016-05-17

**Authors:** M. C. Post, E. E. van der Wall

**Affiliations:** 1 Department of Cardiology, St. Antonius Hospital, Nieuwegein, The Netherlands; 2 Netherlands Society of Cardiology/Holland Heart House, Utrecht, The Netherlands

In this special issue of the Netherlands Heart Journal different aspects of pulmonary hypertension are elucidated.

Pulmonary hypertension (PH) is a haemodynamic state defined as a mean pulmonary artery pressure of ≥25 mmHg assessed by right heart catheterisation at rest [1]. Furthermore, PH can be divided into two main haemodynamic profiles: a pre-capillary (with a pulmonary artery wedge pressure (PAWP) ≤15 mmHg) and post-capillary form (with a PAWP >15 mmHg).

Subsequently, PH can be found in different clinical conditions and is categorised into five groups based on a similar presentation, haemodynamic profile, pathological findings, and treatment: pulmonary arterial hypertension (PAH) (group 1), PH based on left heart disease (group 2), PH due to lung disease and/or hypoxaemia (group 3), chronic thromboembolic PH (CTEPH) (group 4), and PH with unclear and/or multifactorial mechanisms (group 5) [1, 2]. Patients with congenital heart disease (CHD) with a prevalent or corrected systemic-to-pulmonary shunt are at risk for the development of PAH [3]. One of the best examples of a disease associated with PH based on a multifactorial aetiology (group 5) is sarcoidosis, a rare multisystemic disorder characterised by non-caseating granulomas that can present in multiple tissues. Both pre- and post-capillary forms of PH might be found. In a review by Huitema et al. different aspects of PH in pulmonary sarcoidosis are described, underlying the importance of a multidisciplinary approach [4].

The prevalence of PH varies between each group; left-sided heart disease is the most common cause of PH. Up to 70 % of patients with severe chronic heart failure suffered from PH [5], whereas the prevalence of pulmonary arterial hypertension (PAH) is estimated to be between 15 and 60 patients per million population [6]. The estimated prevalence of PAH in CHD is about 10 %, depending on the underlying defect and other characteristics [7]. In all subgroups of PH, quality of life (QoL) is decreased and mortality substantially increased compared with patients without PH. The most important predictors for survival are: functional class or exercise capacity, QoL, performance of the right ventricle and different pulmonary haemodynamic measurements found by right heart catheterisation [1]. The diagnosis of PAH has a tremendous psychosocial impact on patients and their families, and is associated with a high prevalence of anxiety and depression, in up to 40 % with an impairment of QoL [8, 9].

Wapenaar et al. describe the validation of the disease-specific Cambridge Pulmonary Hypertension Outcome Review (CAMPHOR) for the Dutch population [10]. Different standard generic questionnaires for heart and lung diseases have been used to report QoL in PH. However, the CAMPHOR seems to be superior to the standard questionnaires in the assessment of PH-related outcome [11]. Therefore, it is important to use the Dutch version of this questionnaire to have a valid and reliable measure of the QoL in patients with PH.

Transthoracic echocardiography is mandatory as first-line screening tool in patients at risk for the development of PH or if the presence of PH is clinically suspected. Based on the peak tricuspid regurgitation velocity (TRV) and the presence of additional findings (including measurements of the right and left ventricle, pulmonary artery and inferior vena cava or right atrium), the echocardiographic probability of PH is divided into low (TRV <2.8 m/s without additional findings), intermediate or high (TRV 2.9 to 3.4 m/s with additional findings or >3.4 m/s) [1]. Furthermore, echocardiography can be helpful in detecting the cause of PH, mainly associated with left and/or congenital heart disease and seems to predict outcome.

In a review article by Baggen et al., the association between different echocardiographic parameters and outcome, defined as mortality or lung transplantation, was investigated in a meta-analysis [12]. They found that the presence of pericardial fluid, right atrial enlargement, and a decrease in the tricuspid annular plane systolic excursion are the best predictors for worse outcome.

Based on the echocardiographic probability of PH further diagnostic evaluation should be performed to identify the aetiology of PH, including pulmonary function tests, chest CT and ventilation/perfusion scintigraphy to exclude lung disease and pulmonary embolism [1, 13]. If PAH or CTEPH is suspected the patient should be referred to an expert centre to confirm the diagnosis by specific diagnostic tests, including right heart catheterisation. Goal-oriented targeted PH-specific therapy is indicated in symptomatic patients (functional class ≥2) with pulmonary vascular disease, besides general and supportive therapy [1]. The response to this specific medication should be evaluated every 3 to 6 months based on symptoms, functional class, exercise capacity, biomarkers, imaging and sometimes haemodynamics.

Van Riel et al. evaluate the differences in clinical presentation and outcome after the initiation of PH-specific therapy in patients with CHD in the Netherlands versus Singapore [14]. They and others found that the time of clinical presentation, effect and type of initial treatment is influenced by differences in socioeconomic status and healthcare systems [14, 15].

If the clinical response is inadequate sequential double or triple therapy should be given, and maybe other appropriate therapies should be discussed in a multidisciplinary team.

Couperus et al. describe the tailored interventional treatment of complex CHD patients suffering from PH with an impaired pulmonary flow, and emphasised the importance of a multidisciplinary approach [16]. Next to optimal medical PAH-specific therapy, an invasive circulatory adjustment might be necessary to decrease right ventricular overload. Pre-procedural invasive pulmonary haemodynamic measurement (including pulmonary vascular resistance and effective left-to-right shunt quantification) is highly important to evaluate the feasibility of shunt intervention to prevent acute right ventricular failure [17]. Thereafter, each case should be discussed within a multidisciplinary team to reach consensus regarding patient treatment. Therefore, the current guidelines recommend that treatment and follow-up of patients with PAH (and PAH-CHD) should be performed in tertiary centres using a multidisciplinary approach [1, 17].

Several aspects of pulmonary hypertension are highlighted in this special issue. These reviews and original articles can further improve our knowledge of PH and might improve our multidisciplinary care in daily practice.
